# Metabolic syndrome, C-reactive protein and microalbuminuria in a rural Chinese population: a cross-sectional study

**DOI:** 10.1186/1471-2369-14-118

**Published:** 2013-06-02

**Authors:** Liping Jiang, Wen Huang, Yuanbo Liang, Fenghua Wang, Xinrong Duan, Xiaohui Yang, Jiangping Wen, Ningli Wang

**Affiliations:** 1 Department of Nephrology, Beijing TongRen Hospital, Capital Medical University, Beijing, China; 2 Beijing TongRen Eye Center, Beijing TongRen Hospital, Capital Medical University, Beijing, China; 3 Department of Laboratory Medicine, Beijing TongRen Hospital, Capital Medical University, Beijing, China

**Keywords:** Microalbuminuria, Metabolic syndrome, Inflammation, Epidemiology

## Abstract

**Background:**

Microalbuminuria is an early marker of chronic kidney disease (CKD). Previous studies have shown that either metabolic syndrome (MetS) or chronic inflammation is related to renal impairment. The aim of this study was to investigate the association between MetS, C-reactive protein (CRP) and microalbuminuria in a rural Chinese population.

**Methods:**

This was a cross-sectional study using data from the Handan Eye Study. MetS was defined according to the Chinese Diabetes Society (CDS) criteria. CRP levels ≥ 3 mg/L were classified as high CRP. Microalbuminuria was defined as a urinary albumin/creatinine ratio (ACR) of 30–300 mg/g.

**Results:**

We included 4191 subjects aged ≥ 30 years in this analysis. The prevalence of MetS and microalbuminuria in the group was 25.7% and 15.6%, respectively. The odds ratio (OR) of microalbuminuria in subjects with MetS was 1.25 (95% confidence interval (CI): 1.03 − 1.51) compared with those without microalbuminuria. In multivariate logistic regression analysis, high blood pressure (OR 1.36, 95% CI: 1.10 − 1.67) and high fasting blood glucose (OR 1.44, 95% CI: 1.17 − 1.76) were independently associated with microalbuminuria. Subjects with high CRP and MetS had a 1.46-fold greater risk of having microalbuminuria compared with those with low CRP without MetS (95% CI: 1.06 − 2.01).

**Conclusions:**

In this rural Chinese population aged ≥30 years, MetS and microalbuminuria were independently related and the combination of high CRP and MetS was associated with an increased risk of microalbuminuria.

## Background

Chronic kidney disease (CKD) has become a worldwide public health problem and microalbuminuria is an early marker of CKD. Many studies have shown that microalbuminuria is a strong and independent predictor of progressive kidney disease, cardiovascular events and all-cause mortality in diabetic, non-diabetic, hypertensive persons, and even in the general population [1-5]. Therefore, early prevention and treatment of microalbuminuria, by inhibiting the renin-angiotensin system, is generally recommended in high risk patients, such as those with diabetes or hypertension [6]. Furthermore, identification and treatment of the risk factors for microalbuminuria may be an effective approach to prevent adverse outcomes.

Metabolic syndrome (MetS) is a common disorder characterized by abdominal obesity, hypertriglyceridemia, low high density lipoprotein (HDL) cholesterol level, high blood pressure (BP), and high fasting blood glucose level. A previous national cross-sectional study reported that the prevalence of MetS was 15.1% in Chinese adults aged 35–74 years [7]. Further, previous studies have shown that MetS is associated with microalbuminuria [8-10].

C-reactive protein (CRP) has been recognized as a sensitive marker of inflammation. Some cross-sectional studies have found a significant correlation between CRP and microalbuminuria in the general population [11,12]; however, other studies found conflicting results [13,14]. Studies examining the relationship between CRP and microalbuminuria in Chinese populations are rare. Recently, we reported that a high prevalence of microalbuminuria (15.3%) was observed in a rural Chinese population aged ≥ 30 years [15]. The aim of the current study was to investigate the association between MetS, CRP and the risk of microalbuminuria in the same rural Chinese population.

## Methods

### Study population

The Handan Eye Study (HES) was a population-based, cross-sectional study conducted from October 2006 to October 2007, which was designed to survey eye diseases and other health-related problems. Detailed information on this study has been published elsewhere [16]. In brief, residents of Yongnian County, Handan city, Hebei province, China, aged ≥ 30 years were randomly selected using a stratified, clustered and multi-staged sampling technique. Yongnian County is located approximately 500 km south of Beijing and covers 980 km^2^. The total population was 830 000 in 2005; 90% of the population were farmers; and 98% were Han. Thirteen of the 458 villages in Yongnian County were randomly selected to achieve a target sample size of 8653, with 7557 participants considered eligible; 6830 underwent a comprehensive eye examination. Data from 4191 participants aged ≥ 30 years were complete and included in this study. The HES protocol was approved by the Beijing TongRen Hospital Ethical Committee and conformed to the Declaration of Helsinki. All subjects gave written informed consent.

### Data collection

Sociodemographic characteristics (age, sex and education), medical history (hypertension, diabetes and cardiovascular disease or stroke), and lifestyle behavior (smoking status and alcohol intake) were obtained using questionnaires. Height, weight and waist circumference were measured according to standard protocols. Body mass index (BMI) was calculated from weight and height measurements as weight (kg) divided by the square of the height (m^2^). BP was measured twice in a sitting position after at least 5 minutes’ rest. An average of the two readings was then calculated. Blood specimens were collected after an overnight fast of at least 8 hours. Serum creatinine (SCr) was measured using a kinetic Jaffe reaction. Glucose, triglycerides and HDL cholesterol were measured by enzymatic methods using an automatic clinical chemistry analyzer (Olympus AU2700, Tokyo, Japan). High-sensitivity CRP was measured using immunonephelometry with the IMMAGE Immunochemistry System (Beckman Coulter, Inc., Fullerton, CA, USA). Fresh midstream urine was collected in the morning and women who were actively menstruating were excluded from the urine test. Urine albumin concentration was measured by immunoturbidimetry (Audit Diagnostics, Cork, Ireland). Urine creatinine was measured by the same method used for SCr.

### Definitions

MetS was confirmed according to the Chinese Diabetes Society (CDS) criteria when a subject had three or more of the following five components [17]: 1) obesity: BMI ≥ 25 kg/m^2^; 2) fasting blood glucose ≥ 6.1 mmol/L or receiving antidiabetic medication; 3) triglycerides ≥ 1.7 mmol/L; 4) HDL cholesterol < 1.04 mmol/L; and 5) BP ≥ 130/85 mmHg or receiving antihypertensive medication. We also analyzed data on central obesity, defined as waist circumference > 90 cm in men or > 80 cm in women according to the American Heart Association/National Heart, Lung, and Blood Institute Scientific Statement (AHA/NHLBI) [18]. A CRP cutoff of 3 mg/L was used to differentiate high and low CRP groups according to the American Heart Association/Centers for Disease Control (AHA/CDC) consensus [19]. Subjects with clinical inflammation (CRP levels > 10 mg/L) were excluded from this study. Urinary albumin/creatinine ratios (ACR) of 30–300 mg/g and < 30 mg/g were defined as microalbuminuria and normoalbuminuria, respectively. Participants with macroalbuminuria (ACR > 300 mg/g) were excluded. We repeated the analysis using gender-specific definitions of microalbuminuria (17–250 mg/g for men and 25–355 mg/g for women) based on the National Kidney Foundation guidelines [20].

Hypertension was defined as systolic BP ≥ 140 mm Hg or diastolic BP ≥ 90 mm Hg, or receiving antihypertensive medication. Diabetes was defined as fasting blood glucose ≥ 7.0 mmol/L or a self-reported history of diabetes.

### Statistical analysis

All analyses were performed using SPSS software, version 11.5 (SPSS, Inc., Chicago, IL, USA). Data are presented as the mean ± SD for continuous variables and as proportions for categorical variables. Appropriate variables were compared using the unpaired *t* test for continuous data and *χ*^*2*^ test for categorical data. Because the CRP distribution was skewed, variables are expressed as medians and interquartile ranges and were compared using the Mann–Whitney test. We calculated the frequency of the individual MetS components, MetS and high CRP. The association between MetS, CRP and microalbuminuria was analysed using univariate and multivariate logistic regression analysis. The odds ratio (OR) of microalbuminuria was calculated according to the CRP levels and MetS status and a 95% confidence interval (CI) was obtained using a multiple logistic regression model. We also used multivariate linear regression analysis to estimate the association between CRP levels (independent variable) and urinary ACR (dependent variable). All reported *p* values are based on two-sided tests and *p* < 0.05 was considered significant.

## Results

### Baseline characteristics

A total of 4191 participants were studied; 55.4% (n = 2320) were female and the mean age was 51.7 ± 11.1 years. The prevalence of microalbuminuria in this rural Chinese population aged ≥ 30 years was 15.6%. Table 1 shows the baseline characteristics according to the microalbuminuria status. Compared with the normoalbuminuria group, the microalbuminuria group had a greater age; higher waist circumference; history of cardiovascular disease; and higher systolic BP; diastolic BP; fasting blood glucose; triglycerides; and CRP levels. Hypertension, diabetes and female gender were prevalent among subjects with microalbuminuria.

**Table 1:** Basic patient characteristics according to microalbuminuria status

	**Total**	**Normoalbuminuria**	**Microalbuminuria** *	***p *value**
	**n = 4191**	**n = 3538**	**n = 653**	
Age (y)	51.7 ± 11.1	51.1 ± 11.1	54.7 ± 10.7	<0.001
Female, No. (%)	2320(55.4)	1919(54.2)	401(61.4)	0.001
Current smoker, No. (%)	1078(26.0)	929(26.5)	149(23.2)	0.09
Current drinker, No. (%)	774(18.7)	659(18.8)	115(18.0)	0.63
High school education, No. (%)	132(3.2)	110(3.1)	22(3.4)	0.71
Cardiovascular disease, No. (%)	207(4.9)	162(4.6)	45(7.0)	0.01
Stroke, No. (%)	101(2.4)	89(2.5)	12(1.9)	0.32
BMI (kg/m^2^)	24.7 ± 3.7	24.7 ± 3.7	24.7 ± 3.8	0.96
Waist circumference (cm)	87.9 ± 9.6	87.7 ± 9.6	88.8 ± 9.5	0.009
Systolic BP (mm Hg)	139.4 ± 22.1	138.1 ± 21.5	146.4 ± 24.4	<0.001
Diastolic BP(mm Hg)	77.7 ± 12.2	77.3 ± 12.0	80.1 ± 13.2	<0.001
Fasting glucose (mmol/L)	5.8 ± 1.3	5.7 ± 1.2	6.1 ± 1.9	<0.001
Triglycerides (mmol/L)	1.5 ± 1.0	1.5 ± 0.9	1.6 ± 1.3	<0.001
HDL cholesterol (mmol/L)	1.3 ± 0.3	1.3 ± 0.2	1.3 ± 0.3	0.33
SCr (umol/L)	71.4 ± 10.8	71.5 ± 10.7	70.8 ± 11.3	0.11
Diabetes, No. (%)	259(6.2)	195(5.5)	64(9.8)	<0.001
Hypertension, No. (%)	2076(49.5)	1672(47.3)	404(61.9)	<0.001
CRP (mg/L)	0.8(0.4-2.1)	0.8(0.4-2.0)	1.0(0.4-2.3)	0.01
High CRP ^†^, No. (%)	660(15.7)	535(15.1)	125(19.1)	0.01
Obesity, No. (%)	1737(41.4)	1467(41.5)	270(41.3)	0.96
High BP, No. (%)	2723(65.0 )	2233(63.1)	490(75.0)	<0.001
High fasting glucose, No. (%)	818(19.5)	643(18.2)	175(26.8)	<0.001
High triglycerides, No. (%)	1185(28.3)	982(27.8)	203(31.1)	0.08
Low HDL cholesterol, No. (%)	797(19.0)	677(19.1)	120(18.4)	0.65
MetS ^‡^, No. (%)	1076(25.7)	873(24.7)	203(31.1)	0.001

*BMI* body mass index, *BP* blood pressure, *CRP* C-reactive protein, *HDL* high density lipoprotein, *MetS* metabolic syndrome, *SCr* serum creatinine.

* Microalbuminuria was defined as a urinary albumin/creatinine ratio (ACR) of 30–300 mg/g.

^†^ High CRP was defined as a CRP ≥3 mg/dl.

^‡^ MetS was defined by the Chinese Diabetes Society (CDS).

A total of 1076 (25.7%) subjects had MetS; 41.4% were obese; 65.0% had high BP; 19.5% had high fasting glucose; 28.3% had high triglycerides; and 19.0% had low HDL cholesterol. High CRP (≥ 3 mg/L) was observed in 660 (15.7%) subjects. The prevalence of MetS and high CRP was higher in subjects with microalbuminuria than in those with normoalbuminuria (31.1% vs. 24.7%, *p* = 0.001 and 19.1% vs. 15.1%, *p* = 0.01).

### Association between MetS, CRP and microalbuminuria

Table 2 shows the ORs for microalbuminuria by individual MetS component, MetS and high CRP. In the unadjusted regression analysis, high BP (OR 1.76, 95% CI: 1.45 − 2.12) and high fasting blood glucose (OR 1.65, 95% CI: 1.36 − 1.99) were associated with microalbuminuria. After adjusting for age, sex, smoking status, alcohol use, education level, history of cardiovascular disease or stroke and CRP, the associations were still significant. Adjusting for the components of MetS had little effect on the ORs. In the multivariate logistic regression model, MetS was independently associated with microalbuminuria (OR 1.25, 95% CI: 1.03 − 1.51, *p* = 0.02); however, the association between high CRP and microalbuminuria disappeared after adjustments were made for age, sex, and other possible risk factors for CKD (*p* = 0.34). A similar result was observed in the multivariate linear regression analysis; after adjusting for age, sex and the components of MetS, there was no relationship between CRP and urinary ACR (*p* = 0.78).

**Table 2:** Odds ratios for microalbuminuria by individual MetS component, MetS and CRP

	**Unadjusted**		Adjusted model1*		**Adjusted model2**^†^	
	**OR (95% CI)**	***p*value**	**OR (95% CI)**	***p*value**	**OR (95%CI)**	***p*value**
Obesity	1.00(0.84-1.18)	0.96	0.98(0.82-1.17)	0.81	0.92(0.76-1.10)	0.34
High BP	1.76(1.45-2.12)	<0.001	1.38(1.13– 1.70)	0.002	1.36(1.10– 1.67)	0.004
High fasting glucose	1.65(1.36-1.99)	<0.001	1.47(1.20 -1.80)	<0.001	1.44(1.17 -1.76)	<0.001
High triglycerides	1.17(0.98-1.41)	0.08	1.10(0.91-1.32)	0.35	1.03(0.85-1.26)	0.76
Low HDL cholesterol	1.95( 0.77-1.18)	0.65	1.03(0.83 -1.29)	0.77	1.04(0.82 -1.30)	0.76
High CRP	1.33(1.07-1.65)	0.01	1.16(0.93-1.45)	0.19	1.12(0.89-1.40)	0.34
MetS^‡^	1.38(1.15-1.65)	0.001	1.25(1.03 -1.51)	0.02	—	—

*BP* blood pressure, *CRP* C-reactive protein, *CI* confidence interval, *HDL* high density lipoprotein, *MetS* metabolic syndrome, *OR* odds ratio.

* Adjusted for age, sex, smoking status, alcohol use, education level, history of cardiovascular disease or stroke and CRP (except for analyses on the High CRP).

^†^ Additionally adjusted for the other components of MetS.

^‡^ Compared with those without MetS (<3 components).

### Interrelationship between MetS, CRP and microalbuminuria

Table 3 shows the ORs for microalbuminuria by CRP levels and MetS status. The prevalence of microalbuminuria in each group was as follows: low CRP without MetS, 14.2%; high CRP without MetS, 16.3%; low CRP with MetS, 17.6%; and high CRP with MetS, 22.8%. There was no significant difference between the low CRP without MetS and high CRP without MetS groups (14.2% vs. 16.3%, *p* = 0.26) as well as between the low CRP with MetS and high CRP with MetS groups (17.6% vs. 22.8%, *p* = 0.06). Compared with the low CRP without MetS group, the multivariate adjusted OR for microalbuminuria was 1.23 (95% CI: 0.99 − 1.52) and 1.05 (95% CI: 0.78 − 1.42) in the high CRP without MetS and low CRP with MetS groups, respectively. Subjects with high CRP and MetS had a 1.46-fold increase in OR for microalbuminuria (95% CI: 1.06 − 2.01, *p* = 0.02).

**Table 3:** Odds ratios for microalbuminuria by CRP levels and MetS status

	**N**	**Microalbuminuria**	**Unadjusted**		Multivariate-adjusted*	
		**N. (%)**	**OR (95% CI)**	***p*value**	**OR (95%CI)**	***p*value**
Low CRP without MetS	2723	386(14.2)	reference		reference	
High CRP without MetS	392	64(16.3)	1.29(1.05-1.59)	0.02	1.23(0.99-1.52)	0.07
Low CRP with MetS	808	142(17.6)	1.09(0.91-1.26)	0.26	1.05(0.78-1.42)	0.74
High CRP with MetS	268	61(22.8)	1.78(1.32-2.42)	<0.001	1.46(1.06-2.01)	0.02

*CRP* C-reactive protein, *CI* confidence interval, *MetS* metabolic syndrome, *OR* odds ratio.

*Adjusted for age, sex, smoking status, alcohol use, education level, history of cardiovascular disease or stroke.

### Sensitivity analysis

First, we included 4180 subjects with complete information in the analysis of central obesity. The prevalence of MetS and microalbuminuria was 30.1% (n = 1259) and 15.5% (n = 649), respectively and there was a strong and independent relationship between MetS and microalbuminuria after further adjustments (OR 1.40, 95% CI: 1.17 − 1.68, *p* < 0.001).

Second, the results were similar when gender-specific microalbuminuria cutoffs were applied; a total of 1076 (23.2%) subjects had microalbuminuria. In the multivariate-adjusted model, MetS was independently associated with microalbuminuria (OR 1.23, 95% CI: 1.05 − 1.45, *p* = 0.01), and we found no relationship between CRP and microalbuminuria (*p* = 0.14). Subjects with high CRP and MetS had a 1.37-fold greater risk of having microalbuminuria compared with those with low CRP without MetS (95% CI: 1.03 − 1.82, *p* = 0.03).

Third, after excluding subjects with hypertension, the prevalence of MetS and microalbuminuria decreased substantially from 25.7% to 12.8% and from 15.6% to 11.8%, respectively and MetS was not significantly associated with microalbuminuria (OR 1.01, 95% CI: 0.67 − 1.53, *p* = 0.98).

## Discussion

In this rural Chinese population aged ≥ 30 years, we found that MetS, as defined by the CDS, was independently associated with microalbuminuria after adjusting for age, sex and other potential confounding factors. High BP and high fasting blood glucose were the two main risk factors. In addition, we found that CRP was not related to microalbuminuria in the multivariate analysis; however, the combination of high CRP and MetS increased the risk of microalbuminuria.

We used ACR to assess microalbuminuria in our study. The prevalence of microalbuminuria in this rural population was 15.6%, higher than other population-based studies in the US (6.4% in 5659 adults aged 20–80 years in the National Health and Nutrition Examination Survey (NHANES III)) [8], Japan (13.7% in 2321 participants aged 40–87 years) [9] and China (5.3 − 11.5%) [10,21-24]. Notably, the reports from Chinese urban regions showed that the prevalence of microalbuminuria was 5.3% in Beijing (2310 adults aged ≥ 40 years) [21]; 5.8% in Guangzhou (6311 adults aged ≥ 20 years) [22]; 6.7% in Shanghai (3532 adults aged ≥ 20 years) [23]; 8.8% in Hangzhou (2969 adults aged 18 − 87 years) [24]; and 11.5% in Taiwan (2311 adults aged ≥ 40 years) [10]. The discrepancy may be due to differences in the sample size and population studied, including factors such as age, race, region or other factors.

The prevalence of MetS in our study was 25.7%, lower than two urban population studies in Beijing (34.1%) [21] and Taiwan (39.1%) [10], and higher than a recent study in Hangzhou (12.6%) [24]. The difference between urban and rural populations may be partly due to different age groups, geographic regions or definitions of MetS. Our study identified a positive relationship between MetS and microalbuminuria in the rural Chinese adult population, which was compatible with previous studies. Nevertheless, the association between the specific MetS components and microalbuminuria in these populations was inconsistent. Studies in the US and Japan reported that high BP, high fasting blood glucose and obesity were associated with microalbuminuria [8,9]. The results from two Chinese studies demonstrated that all MetS components were associated with microalbuminuria [10,24]. In our study, high BP and high fasting blood glucose were independent risk factors for microalbuminuria, and no association between other MetS components and microalbuminuria was seen. A similar result was obtained in a Chinese urban population study [23]. High BP and high fasting blood glucose are two important components of MetS. As previously reported, our study revealed that mildly elevated BP (≥ 130/85 mm Hg) or fasting blood glucose levels (≥ 6.1 mmol/l) were independently associated with microalbuminuria. Based on these findings, we can easily identify subjects who are at risk of microalbuminuria by measuring BP and fasting blood glucose level. In addition, sensitivity analysis revealed that the observed correlation between MetS and microalbuminuria was strongly affected by the presence of hypertension, which may be attributed to the high prevalence of hypertension (49.5%) in the present study, higher than previous reports from Beijing (47.1%) [21] and southern China (19.2%) [22]. The reason for this may be the different age groups, cardiovascular risk factors or lifestyle and dietary patterns which could potentially affect blood pressure. Hypertension may be an important threat for microalbuminuria in this rural population.

Although several epidemiological studies have confirmed the relationship between MetS and microalbuminuria, the underlying mechanisms are still not completely understood. Several possible reasons have been proposed, including insulin resistance, inflammation, renal endothelial dysfunction, altered renal hemodynamics, and others [25]. The results of our and other studies have shown that CRP, a widely used marker of chronic inflammation, was strongly related to MetS [26-29]. Data from population-based studies from the US [11], Japan [12] and Singapore [30] have shown that CRP was associated with microalbuminuria. These findings suggest that inflammation may play an important role in early kidney damage. However, even though 15.7% of subjects had high CRP levels (≥ 3 mg/L) in our study, a relationship between high CRP and microalbuminuria was not seen. This is similar to previous reports showing that CRP was associated with decreased renal function but not albuminuria [13], or that microalbuminuria was related to a different marker of inflammation (e.g., fibrinogen) but not CRP in a logistic regression model [14]. We also examined the interrelationship between CRP and MetS and the results showed that only the combination of high CRP and MetS was independently associated with microalbuminuria (OR 1.46, 95% CI: 1.05 − 2.01) after further adjustments. Our findings are supported by a recent study indicating that the odds for CKD (defined as an estimated glomerular filtration rate (eGFR) < 60 ml/min/1.73 m^2^) increased only in the subgroup having both high CRP and MetS [31]. In another study, CRP was associated with CKD (defined as an eGFR < 60 ml/min/1.73 m^2^ or albuminuria) independent of MetS, and subjects with high CRP and MetS had the highest odds for CKD of the four groups [32]. These results suggest a positive interrelationship between these MetS and CRP, and microalbuminuria. One consideration is that inflammation and MetS had synergistic or additive effects on renal damage.

This study had a number of limitations. First, a causal relationship cannot be established between MetS and microalbuminuria because of the cross-sectional study design. Second, the ACR measurement was based on a single spot urine sample, which could result in misclassification of the microalbuminuria status. However, this simple sampling procedure was commonly used in epidemiologic studies. Third, several studies have reported that statin drugs may decrease the CRP levels [33,34]. We did not control for the effect of statin drugs in this study, because the number of statin users was relatively small. Only 1.7% (n = 71) of participants answered whether lipid-lowering drugs were being taken during the study period. Finally, selection bias might, in theory, have influenced our results.

## Conclusions

In conclusion, our study showed that MetS was associated with microalbuminuria in a rural Chinese population aged ≥ 30 years. Despite a lack of an independent association between CRP and microalbuminuria, high CRP combined with MetS increased the risk of microalbuminuria. Further prospective cohort studies are necessary to clarify these causal relationships.

## Abbreviations

ACR: Albumin/creatinine ratio; BMI: Body mass index; BP: Blood pressure; CDS: Chinese Diabetes Society; CI: Confidence interval; CKD: Chronic kidney disease; CRP: C-reactive protein; eGFR: Estimated glomerular filtration rate; HDL: High density lipoprotein; MetS: Metabolic syndrome; OR: Odds ratio; SCr: Serum creatinine.

## Competing interests

The authors declare that they have no competing interests.

## Authors' contributions

NW, YL, FW, XD, XY, LJ and WH participated in the study design. YL, FW, XD, XY and LJ collected the data. JW performed the CRP assays. LJ and WH contributed to the data analysis and manuscript preparation. All authors read and approved the final manuscript.

## Pre-publication history

The pre-publication history for this paper can be accessed here:

http://www.biomedcentral.com/1471-2369/14/118/prepub
